# Variation in the Elastic Modulus and Increased Energy Dissipation Induced by Cyclic Straining of *Argiope bruennichi* Major Ampullate Gland Silk

**DOI:** 10.3390/biomimetics8020164

**Published:** 2023-04-18

**Authors:** Ping Jiang, Lihua Wu, Menglei Hu, Sisi Tang, Zhimin Qiu, Taiyong Lv, Manuel Elices, Gustavo V. Guinea, José Pérez-Rigueiro

**Affiliations:** 1 Key Laboratory for Biodiversity Science and Ecological Engineering, Institute of Eco-Environment and Resources, College of Life Sciences, Jinggangshan University, Ji’an 343009, China; 2 Institute of Qinghai-Tibetan Plateau, Southwest Minzu University, Chengdu 610041, China; 3 Department of Nuclear Medicine, Affiliated Hospital in Southwest Medical University, Sichuan Key Laboratory of Nuclear Medicine and Molecular Imaging, Luzhou 646000, China; 4 Departamento de Ciencia de Materiales, ETSI Caminos, Canales y Puertos, Universidad Politécnica de Madrid, 28040 Madrid, Spain; 5 Biomedical Research Networking Center in Bioengineering, Biomaterials and Nanomedicine (CIBER-BBN), 28029 Madrid, Spain; 6 Biomaterials and Regenerative Medicine Group, Instituto de Investigación Sanitaria del Hospital Clínico San Carlos (IdISSC), C/Prof. Martín Lagos s/n, 28040 Madrid, Spain; 7 Center for Biomedical Technology (CTB), Universidad Politécnica de Madrid, Pozuelo de Alarcón, 28223 Madrid, Spain

**Keywords:** spider silk, mechanical properties, viscoelasticity, toughness

## Abstract

The trends exhibited by the parameters that describe the mechanical behaviour of major ampullate gland silk fibers spun by *Argiope bruennichi* spiders is explored by performing a series of loading-unloading tests at increasing values of strain, and by the subsequent analysis of the true stress-true strain curves obtained from these cycles. The elastic modulus, yields stress, energy absorbed, and energy dissipated in each cycle are computed in order to evaluate the evolution of these mechanical parameters with this cyclic straining. The elastic modulus is observed to increase steadily under these loading conditions, while only a moderate variation is found in the yield stress. It is also observed that a significant proportion of the energy initially absorbed in each cycle is not only dissipated, but that the material may recover partially from the associated irreversible deformation. This variation in the mechanical performance of spider silk is accounted for through a combination of irreversible and reversible deformation micromechanisms in which the viscoelasticity of the material plays a leading role.

## 1. Introduction

Spider silk exhibits a series of desirable properties that thrust an intensive research activity led to create new materials with novel properties that may substitute many of the artificial polymeric fibers presently used [1,2,3,4]. Thus, it is usually highlighted that the combination of high tensile strength and strain at breaking characteristic of major ampullate gland silk (MAS) yields values of the work to fracture that surpass those found in any other natural or artificial material [5,6]. Additionally, spider silk is also analysed due to its excellent biocompatibility and its ability to be processed into different formats such assponges, films, and fibers [7,8,9,10]. Furthermore, a number of studies focused on the integrity and robustness of spider webs has highlighted the role of the nonlinear behaviour of these fibers in comparison with other common, mostly artificial, materials [11,12]. An increasing knowledge on all these aspects of spider silk may be invaluable to propose new alternatives for the application of bioinspired perspectives in structural engineering based on such concepts as compartmentalization and complexity [13].

In addition to these features, MAS fibers are also endowed with the ability to recover their properties independently of their previous loading history through the process of supercontraction [14]. Supercontraction was initially identified by the significant shrinkage of the fiber when immersed in water or exposed to a high relative humidity environment [15,16]. Although it was initially considered as a curiosity of spider silk, it was later found that supercontraction plays a major role in the mechanical performance of the material [17,18] and, as indicated above, offers the possibility of reverting the irreversible deformations induced in the fibers when subjected to tensile stresses. Besides, the characterization of the mechanical properties from maximum supercontracted fibers allows the removal of most of the variability usually found in natural materials [19]. In this context, the application of the basic design principles that underpin the behaviour of spider silk and, in particular, of its toughness, nonlinear behaviour, and the existence of supercontraction, should lead to a new generation of load-bearing materials that, in turn, would be the critical elements of more robust and efficient structures.

Paradoxically—and in spite of recognizing the variation in the properties of the material when tested in air that results from the action of the various deformation micromechanisms that determine the behaviour of the fibers—these changes in the tensile behaviour of spider silk have not been characterized in detail. This work is intended to identify the basic trends exhibited by the parameters that define the mechanical behaviour of MAS silk subjected to a series of loading-unloading cycles. It must be recognized, however, that more extensive studies will be required in order to obtain a truly predictive and quantitative theory of this variation. In this context, the effect of cyclic straining in the elastic modulus, the yield stress, and the ability of the material to absorb and dissipate mechanical energy are considered and discussed.

## 2. Materials and Methods

MAS fibers of *Argiope bruennichi* (Scopoli, 1772) spiders were used in this work. Spiders were bred in 60 cm × 60 cm × 60 cm cages and fed a diet of houseflies. Silk fibers were forcibly silked [20] by pulling the fiber from the spider at a controlled speed of 1 cm s^–1^ at 20 °C and 35% relative humidity (RH). The spider was not anaesthetized at any step of the process. Samples were stored at nominal conditions 20 °C and 35% RH until testing.

Silk samples were mounted on aluminium foil frames as described elsewhere [21] The gauge length of the samples was 20 mm, and the cross-sectional areas of the forcibly silked fibers were calculated from optical and/or scanning micrographs, taking into account the circular cross section observed in MAS fibers, as previously described [22]. Some examples of the SEM micrographs used to calculate the cross-sectional area are presented in Figure S1. The samples were subjected to maximum supercontraction [23] by being immersed in water for 30 min. Samples were allowed to dry overnight before being tensile tested. The cross-sectional area of the samples after maximum supercontraction (*A*_0_) was calculated from the cross-sectional area measured on the forcibly silked fibers (*A_FS_*) by applying the conservation of the volume in spider silk as:

(1) $$A_{0}=\frac{A_{FS}L_{FS}}{L_{0}}$$

where *L_FS_* is the initial length of the forcibly silked fiber, and *L*_0_ is the length of the fiber after supercontraction. The conservation of volume is a characteristic property of spider silk assessed by measuring the diameters of fibers subjected to different elongations [24], and is conventionally applied for calculating the mechanical parameters of MAS. The values of the cross-sectional area, *A*_0_, and of the length, *L*_0_, after supercontraction were used for the calculation of the stresses and strains of the sample.

**Figure 1:**
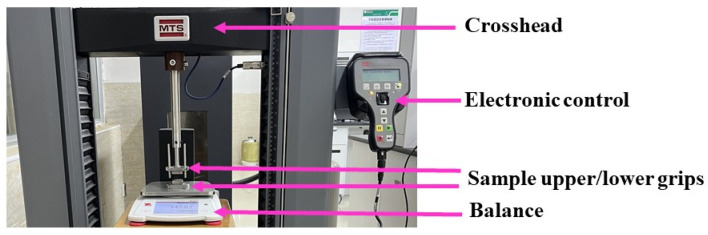
Experimental setup used for the tensile tests.

Tensile tests were performed on three different maximum supercontracted MAS fibers in an MTS (E44.104, Eden Prairy, MN, USA) electric tensile testing machine, as illustrated in Figure 1. Although statistically the usage of forcibly silked fibers reduced the typical variability found in spider silk fibers [25,26], since spiders tend to exert high spinning stress on the fiber during this process, the usage of maximum supercontracted fibers is intended to fully eliminate the variability in the mechanical behaviour typically associated with silk fibers [14]. All tensile tests were performed in air at a strain rate of 10 mm/min (which corresponds to, approximately, a strain rate of 0.5/min) under nominal environmental conditions 20 °C and 35% relative humidity. Tensile testing consisted of a series of loading-unloading cycles as illustrated in Figure 1. Nominally, in each cycle the sample was to be stretched up to a strain value 0.025 greater than the maximum strain value reached in the previous cycle. However, upon performing the testing some deviations may have occurred from this nominal procedure, so that the actual increase in strain of a given cycle may be occasionally lower or greater than the nominal value of 0.025. Upon reaching this increment in strain, the sample was unloaded at 10 mm/min until the distance between the grips of the tensile testing machine corresponded to the initial length of the sample after supercontraction, *L*_0_. The sample was allowed to relax for 20 min while maintaining the grips of the tensile testing machine at a distance *L*_0_, before starting a new cycle.

Engineering stress, *s*, and engineering strain, *e*, were calculated from the measurements of force and displacement of the test using the initial values of the cross-sectional area, *A*_0_, and length, *L*_0_, as:

(2) $$s=\frac{F}{A_{0}}\;;\;e=\frac{\Delta L}{L_{0}}$$

where *L* corresponds to the increase in length of the sample that results from tensile testing. Since the compliance of the fiber is much larger than that of the rest of the experimental setup, as observed in Figure 1, where the main, mostly metallic, elements that compose this setup are shown, this value is usually approximated by the displacement of the crosshead [21], so that the usage of an extensometer is not required.

**Figure 2:**
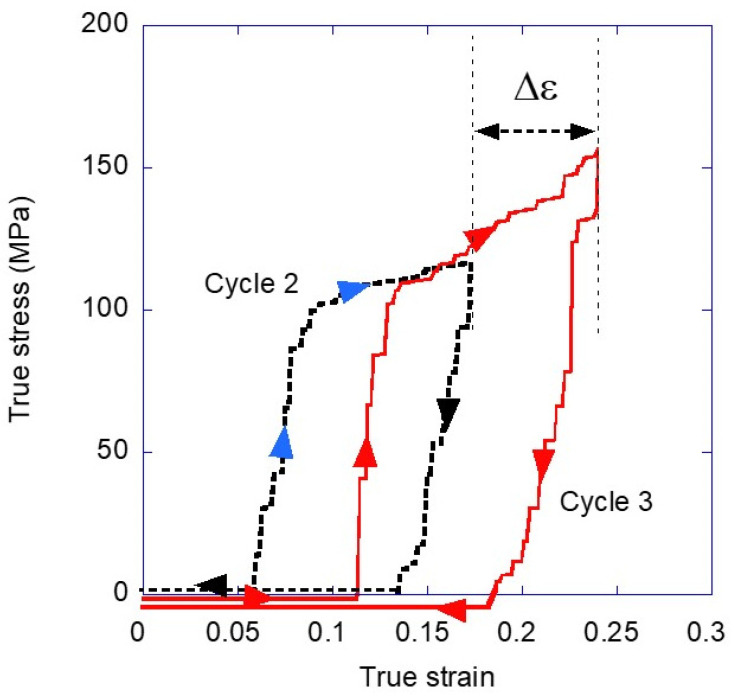
Scheme of the loading-unloading cycles. The lines of part of the unloading and reloading steps are drawn below the zero value of force to prevent their overlapping and to illustrate the whole process more clearly. All these lines correspond to a value of *σ* = 0 MPa.

The process followed to obtain the true stress-true strain is illustrated in Figure 2 using the cycles 2 and 3 as representatives. The sample is loaded (blue arrowheads) up to a given true strain (*ε* = 0.17 for cycle 2) and unloaded (black arrowheads), until it reaches a true strain of *ε* = 0. The fiber is allowed to relax for at least 20 min at this value of true strain before starting the new cycle (cycle 3, red arrowheads). True stress, *σ*, and true strain, *ε*, were calculated from the engineering magnitudes by using the hypothesis of constant volume [24] as:

(3) $$\sigma =s\cdot \left(1+e\right)\;;\;\varepsilon =Ln(1+e)$$

**Figure 3:**
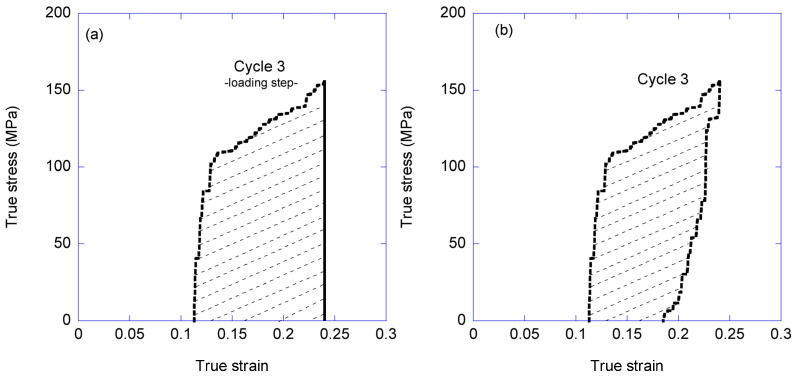
Scheme of the energy absorbed and dissipated in a loading-unloading cycle. (**a**) Scheme of the calculation of the absorbed energy in a single cycle, which is defined as the area below the true stress-true strain curve from the initial true strain value at which *σ* = 0 up to the maximum value of the true stress in the cycle (dashed area in **a**). (**b**) Scheme of the calculation of the dissipated energy in a single cycle defined as the area enclosed between the curves of the loading and unloading steps of the cycle (dashed area in **b**).

The true stress-true strain curves were also used to calculate the absorbed and dissipated energies in a cycle. The absorbed energy is defined as the area below the stress-strain curve up to the maximum strain reached in that cycle. The dissipated energy is calculated as the area enclosed by the loading and unloading curves as illustrated in Figure 3, once again using cycle 3 as an example.

## 3. Results

**Figure 4:**
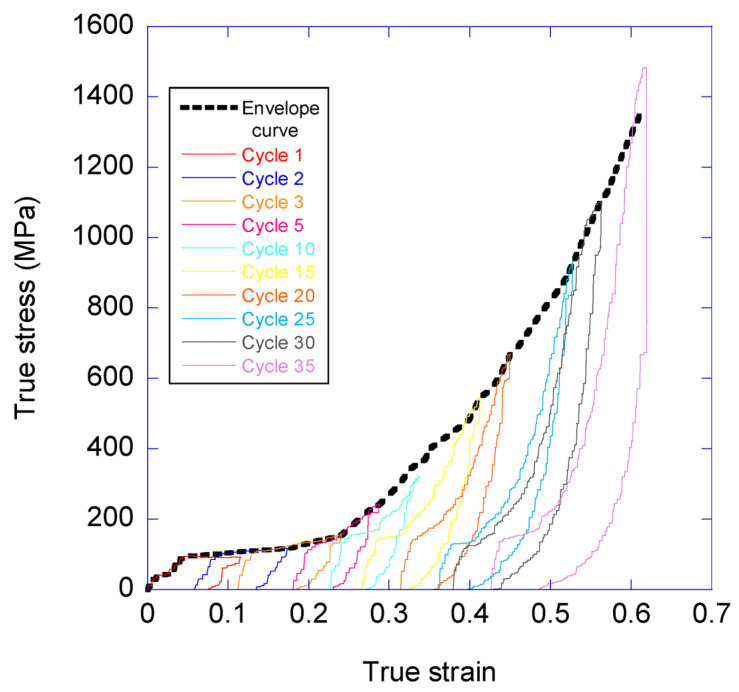
True stress-true strain curves of several selected loading-unloading cycles obtained from a single fiber. The envelope curve (broken line) is drawn by interpolating the values between the maximum values of the true stress reached in each cycle. The same data represented as engineering stress vs. engineering strain may be found in Figure S3.

Three MAS samples were tested and found to show comparable tensile behaviours. The complete set of loading-unloading cycles measured from the three samples, represented as true stress-true strain curves, is presented as Figure S2a–c. Subsequent analysis of the trends observed in the various mechanical parameters proceeded in detail on the sample that yielded the highest value of tensile strength and strain at breaking, which corresponds to the curves shown in Figure S2a, and comprises 35 loading-unloading cycles on a single fiber. Some selected loading-unloading cycles obtained from this sample are shown in Figure 4. The same data, presented as engineering strain vs. engineering stress, are shown in Figure S3.

**Figure 5:**
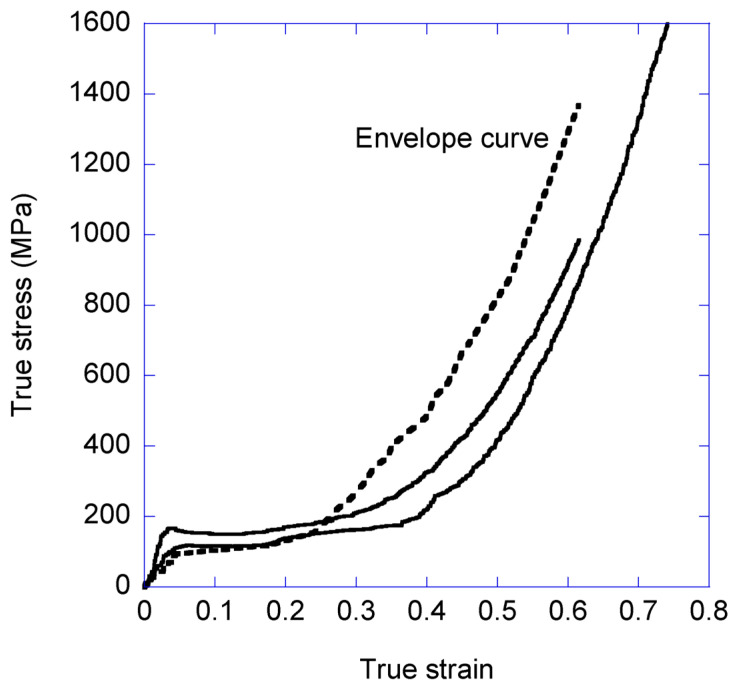
Comparison of the envelope curve and true stress- true strain curves obtained from conventional tensile tests. The envelope curve is determined by interpolating the data of the loading-unloading cycles (broken line) and compared with curves obtained by tensile testing maximum supercontracted *A. bruennichi* fibers (solid lines).

The representation of the true stress-true strain curves of the different cycles allows determining an envelope curve produced by interpolating the values of the maximum stresses reached in all the cycles. The envelope curve obtained from the loading-unloading cycles is compared with the tensile properties of a maximum supercontracted fiber tensile tested until breaking in Figure 5. It is apparent that the true stress-true strain curves recorded from conventional tensile tests on single fibers are qualitatively similar to the envelope curve determined from Figure 4.

In order to obtain a more quantitative confirmation of this similarity, it is possible to calculate an effective α* parameter [27] from the envelope curve and to compare this value with that obtained from conventional tests on supercontracted *A. bruennichi* MAS fibers. The value of the effective value for the envelope curve is found to be α* = 0.49 (Figure S4) and that of the value obtained from conventional tests is α* = 0.41 (the values of the α* parameter are available at http://www.ctb.upm.es/core-facilities/ accessed on 31 March 2023), which further supports the similarity between the curves. The α* parameter is mainly used to classify the tensile behaviour of MAS fibers with a single value [27] and, in this case, it is termed “effective” for the envelope curve, because the α* parameter is properly calculated from conventional tensile tests on maximum supercontracted single fibers. In order to calculate the α* parameter, the curve of interest is displaced along the true strain axis (*X*-axis) until two conditions are fulfilled: (1) the values of the true stress of both the reference and the displaced curve concur, and (2) the slopes of both curves at the point of concurrence differ by less than 20%. The fulfilment of these two conditions is seen to ensure the similarity of both the curve of interest and the reference curve from the point of concurrence up to the strain at breaking.

In the present context, the similarity of the envelope curve and those obtained from conventional tensile tests indicates that the variation in the mechanical properties of the fiber induced during stretching from a given value of true strain onwards is largely independent of the previous loading history of the fiber, at least within the range of strain rates and relaxation time lapses between consecutive cycles employed in this work. In this regard, the choice of 20 min as the lapse time between cycles is intended to remove any possible transient effect due to the viscoelastic behaviour of the fiber, and to allow measuring the proper stationary state of the system in each cycle.

Although it may be possible to expand the concept of α* parameter to characterize individual fibers, even if not subjected to maximum supercontraction [28], in order not to lead to possible confusion we prefer to restrict its usage in this work to the comparison of the mechanical properties exhibited by different fibers, and to describe the evolution of a single fiber during tensile testing with the conventional use of the true strain.

The analysis of the individual loading-unloading cycles also allows determining the effect that previous cycles exert on the variation of the mechanical properties of the material. Among these properties, the possible variation of the elastic modulus, of the yield stress and of the energy absorbed and dissipated by the material, is considered in detail below.

### 3.1. Variation of the Elastic Modulus and Yield Stress with the Loading-Unloading Cycles

**Figure 6:**
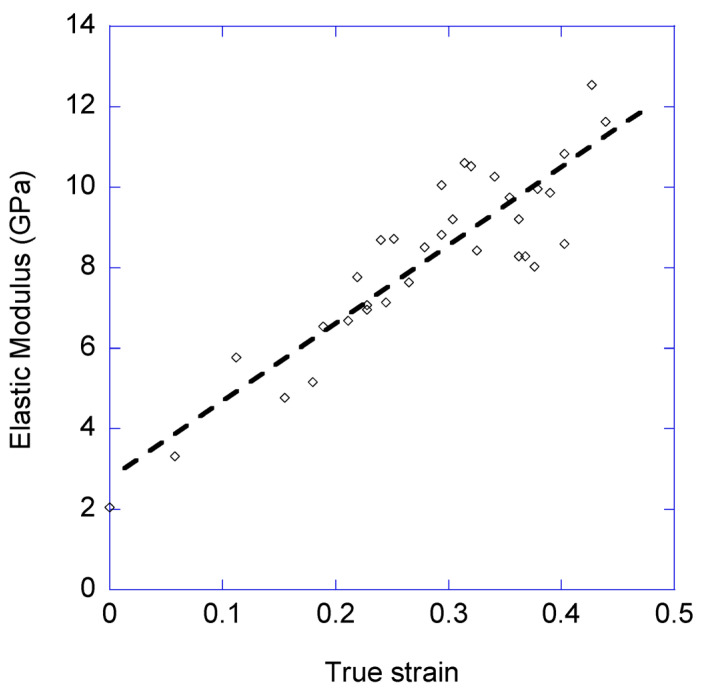
Evolution of the elastic modulus with the loading-unloading cycles. The *X*-axis corresponds to the initial true strain of the cycle (i.e., the true strain at which the fiber starts to be subjected to a load different from zero). True strain is always calculated taking the length of the maximum supercontracted fiber as reference. The broken line corresponds to the least square fitting of the data.

The elastic modulus of the loading curve for each cycle was calculated from the initial slope of the curve. Figure 6 shows the values of the elastic modulus, calculated from the true stress-true strain curves, for all the cycles as a function of the initial true strain of the cycle (i.e., the value of true strain at which the fiber starts to be subjected to a load different from zero).

**Figure 7:**
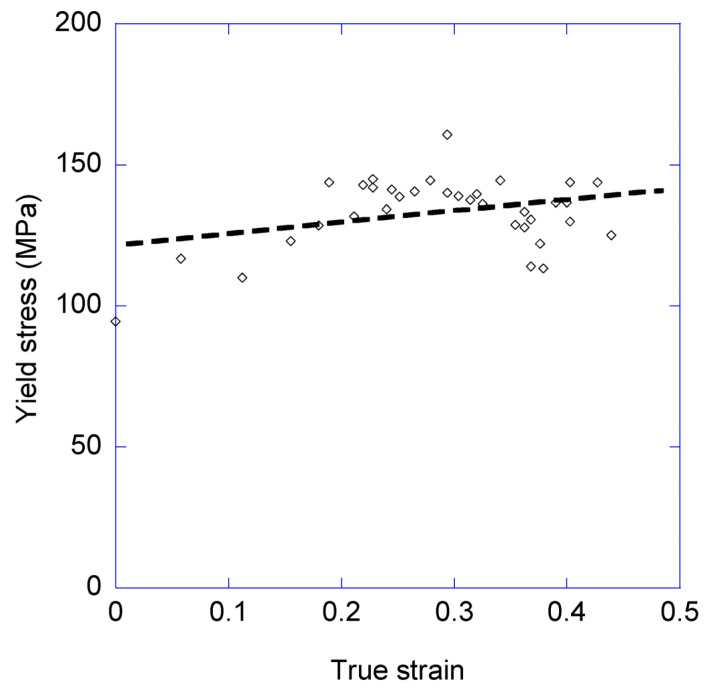
Evolution of the yield stress with the loading-unloading cycles. As in Figure 6, the *X*-axis corresponds to the true strain at which the load on the fiber starts to increase in the cycle. The broken line corresponds to the least square fitting of the data.

In addition, the variation of the yield stress, as determined from the true stress-true strain curves, with the cycles was also calculated and is presented in Figure 7. The yield stress corresponds to the end of the elastic regime of the material, and is defined in this case by the intersection point of the loading curve of the cycle and a straight line with a slope equal to 95% of the elastic modulus calculated for that cycle. This definition of the yield point is sometimes referred to as the proportional limit of the material [29].

Figure 6 shows a clear increase in the elastic modulus with the number of cycles. Using the values obtained from the least squares fitting method, the elastic modulus varies from an initial value of E (cycle 1) ≈ 3 GPa to a value of E (cycle 35) ≈ 12 GPa. In contrast, the yield stress shows a much more moderate variation between the initial value of σ_y_ (cycle 1) ≈ 120 MPa to a value of σ_y_ (cycle 35) ≈ 140 MPa. In this regard, and although the values of the yield stress are fitted in Figure 7 to a straight line for consistency with the analysis of the data corresponding to the elastic modulus, the large variability observed prevents associating such a clear trend to this mechanical parameter as that previously found for the elastic modulus.

### 3.2. Energy Absorption and Dissipation with the Loading-Unloading Cycles

As indicated above, the ability to absorb and dissipate the energy of MAS fibers underpins most of the biological functions of this material. The basic parameter that defines the energy absorbed by the fiber during its deformation is calculated as the work to fracture, W_f_, and corresponds to the area below the stress-strain curve. If the envelope curve is used in this case to estimate the work to fracture that the fiber may absorb in a conventional tensile test (i.e., with no loading-unloading cycles), a value of W_f_ ≈ 270 MJ/m^3^ is obtained. Although other spiders may reach even higher values of work to fracture [6], this value still exceeds those yielded by high performance artificial materials, such as Kevlar 49, with 50 MJ/m^3^ [30].

The analysis on the absorption and dissipation of energy by spider silk fibers may be expanded by using the data obtained from the loading-reloading tests. In particular, the dissipated energy, which is computed as the area enclosed between the loading and unloading curves, corresponds to the fraction of the energy that is initially absorbed, but not stored in the material. The difference between absorption and dissipation of energy is made clearer by considering a material such as rubber at not too large a strain, in which the loading and unloading steps of the stress-strain curve concur. In this case, the material may absorb a significant amount of energy (the area below the stress-strain curve during the test), but this energy is not dissipated, but stored in the material, and may be recovered during the unloading step. Consequently, the dissipation of a large fraction of the absorbed energy will result from a significant difference between the loading and unloading steps of the stress-strain curve in a cycle.

**Figure 8:**
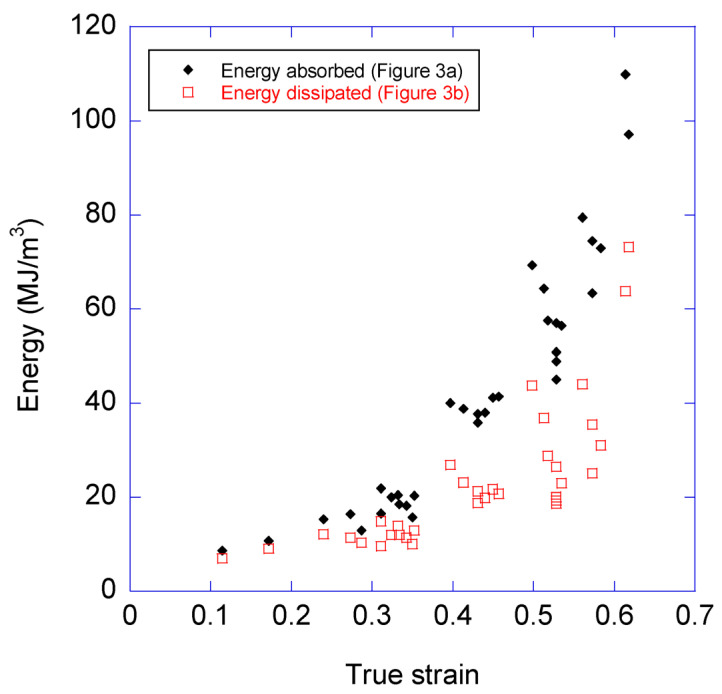
Energy absorbed and dissipated in each cycle. The energy absorbed and dissipated in each cycle is represented by solid black diamonds and by open red squares, respectively. The *X*-axis corresponds to the maximum value of true strain reached in the cycle.

The evolution of the energy absorbed and dissipated in each cycle is presented in Figure 8. The *X*-axis of Figure 8 corresponds to the maximum value of the true strain reached in the corresponding cycle. As expected from the definition used for the absorbed and dissipated energies, the value of the former is always higher than that of the latter, although it is apparent that a large fraction of the absorbed energy is eventually dissipated at all values of true strain.

**Figure 9:**
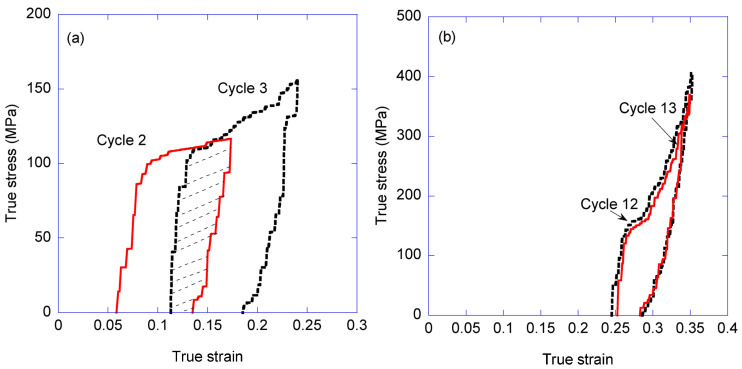
Recovery of the tensile properties between consecutive cycles. (**a**) The value of the maximum true strain reached in the second cycle (cycle 3 in this example) is larger than that of the first cycle (cycle 2 in this example). It is observed that part of the energy absorbed during cycle is irreversibly dissipated, while a significant fraction may be recovered (dashed region between the loading step of cycle 3 and the unloading step of cycle 2). (**b**) The maximum value of true strain reached in the second cycle is similar to that of the previous cycle. In this example, the stress-strain curves of both the loading and the unloading steps concur and the energy is almost completely recovered.

The discrepancy between the value of the energy absorbed in a single conventional tensile test and that calculated by adding the energy dissipated in all the cycles is explained by the ability of the fiber to recover, at least partly, after being unloaded and allowed to relax. This property is illustrated in Figure 9a, once again using cycles 2 and 3 as examples. From Figure 9a it is apparent that the loading step of cycle 3 starts at a lower true strain than that reached at the end of the unloading step of cycle 2. Consequently, the area enclosed by the unloading step of cycle 2 and the loading step of cycle 3 corresponds to the energy that the material recovers. In contrast, Figure 9b illustrates the extreme case in which the loading-unloading curves of two consecutive cycles concur, which implies that all the energy dissipated in the first cycle is again available to the material at the beginning of the second cycle. It should be highlighted that this behavior appears independently of the number of cycles, and depends on reaching a maximum true strain in a cycle that is larger than the one reached in the previous step.

## 4. Discussion

As shown below, the analysis of the evolution in the mechanical parameters of MAS fibers subjected to loading-unloading tests allows establishing correlations between the design principles of this material [31] and its mechanical behavior. To begin, it was shown above that the stress-strain curve of a MAS fiber beyond a given value of true strain (or, equivalently, beyond a given value of true stress) is independent from the previous loading history of the sample, if that value had not been reached previously. This independence correlates well with the concept of hidden length [32,33,34,35] as a key element in the design of these fibers.

The concept of hidden length implies that segments of the protein chains, originally in the amorphous phase, become progressively unfolded during stretching, but until a significant unfolding occurs, those segments do not play any relevant role in the mechanical performance of silk. In this regard, studies based on the Molecular Dynamics of the polyglycine fragments characteristic of the amorphous phase show that these fragments display an elastomeric behaviour [36], independently from the detailed conformation of the participating amino acids. Beyond a given value of true strain (or, correspondingly, of true stress) at which the unfolding of a fraction of those segments initially included in the hidden length takes place, the properties of the material are essentially determined by the behavior of this fraction of the chains. The role of the hidden length fraction in the mechanical behaviour explains why the tensile properties are not affected by the previous history of the sample in which this fraction of the chains, initially in the hidden length, was not involved.

The role of the fraction of protein chains that initially constitute the hidden length may be also recognized in the variation of the mechanical parameters that the fiber undergoes while being stretched. This variation, however, is not immediately apparent in a conventional tensile test, but may be identified from the loading-unloading cycles. In this regard, it is observed that the elastic moduli of the loading steps exhibit a significant variation with increasing values of true strain. This variation implies a monotonous increase in the elastic modulus from an initial value of approx. E = 3 GPa for the maximum supercontracted fiber to a value of approx. E = 12 GPa for the largest values of true strain. This range of elastic moduli is consistent with values found from the mechanical characterization of silks spun by other species [37,38,39].

In this regard, and as mentioned above, this variation of the elastic modulus is consistent with the accepted model of the microstructural organization of spider silk fibers and the underlying deformation micromechanisms [40,41,42]. Spider silk is modelled as a particle-reinforced composite material that is made up of three basic elements [43,44]: a nanocrytalline phase [45] and an amorphous phase that, in turn, comprises two interpenetrating networks of hydrogen bonds [46] and of chains with an elastomeric behavior [47]. The nanocrystalline phase consists of β-nanocrytals that are formed by the piling up of β-pleated sheets [48,49] of those regions of the spidroins that contain the polyalanine motif [50,51]. It was found that the orientation of the β-nanocrystals with respect to the macroscopic axis of the fiber and the crystalline fraction vary among the various spider species [41]. Fewer details are known on the microstructural organization of the amorphous phase [52,53], although it is assumed that the properties of spider silk and, in particular, its work to fracture, depend largely on this phase. In this context, the concept of hidden length defined above would correspond to those segments of the protein chains with elastomeric behavior that are not subjected to a significant load at a given instant.

Based on the previous discussion, the monotonous increase of the elastic modulus with increasing values of true strain may be explained by the interplay between these microstructural elements, and by the deformation micromechanisms in which these elements participate. Thus, a combined microstructural study applying both X-ray diffraction and Raman spectroscopy to *Argiope trifasciata* MAS fibers subjected to increasing strains established that the polyalanine β-nanocrytals increase their alignment with the macroscopic axis of the fiber in the initial stages of a conventional tensile test [54]. When the chains that form the polypeptide backbone become aligned with the macroscopic axis of the fiber, a second deformation micromechanism was identified that implies an increase in the volume fraction of the crystalline phase. In this initial study it was not possible to identify the detailed mechanism that leads to the increase of the crystalline volume fraction, but subsequent results obtained from X-ray diffraction studies on flagelliform silk fibers showed that this deformation micromechanism may consist in the growth of the nanocrystals due the accretion of stretched portions of segments initially in the amorphous regions [55] to the ends of the polyalanine β-nanocrystals. Numerical simulations on the sequence of MAS spidroins also reflect an increase in crytallinity due to the organization of regions initially found in the hidden length [33] during tensile testing. The combination of both mechanisms, the orientation of the nanocrystals until the covalent backbone becomes aligned with the macroscopic axis of the fiber, and the increase of the volume fraction of the crystalline phase, explains the monotonous increase observed in the elastic modulus with increasing values of true strain.

In contrast, no significant variation is observed in the value of the yield stress, which exhibits a remarkable constancy in the range between 120 MPa and 140 MPa. This result can also be accounted for by using the model mentioned above, and assuming that the yield stress results from the breaking of the hydrogen bonds that contribute to the initial hydrogen bond network [43,44]. Under this hypothesis, the value of the yield stress may be assumed to be largely independent from the unfolding of the segments initially included in the hidden length, as observed experimentally.

As a final remark, the singular performance of MAS spider silk in terms of its ability to absorb and dissipate mechanical energy may be traced back to the viscoelastic behavior of the material. Although experimental difficulties usually hamper obtaining detailed information on the viscoelastic behavior of spider silk [56,57,58], it was found that these fibers have the ability to dampen torsional oscillations efficiently, while eventually recovering their form without any external stimuli [59]. This property was discussed as a kind of shape memory in connection with a comparable performance observed in some metallic alloys [60]. A similar mechanism may account for the partial recovery of the properties of the fiber after being allowed to relax, as found from the loading-unloading cycles.

**Figure 10:**
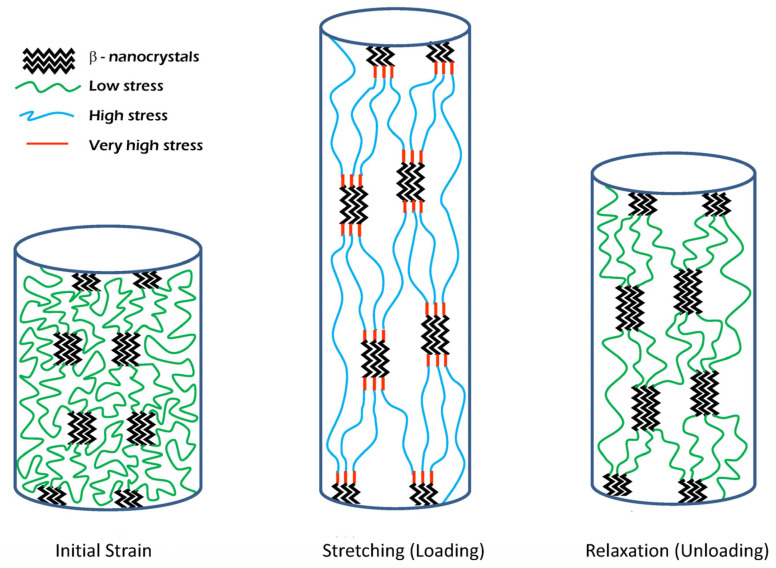
Scheme of the interplay between the different microstructural elements that appear in MAS fibers during a loading-unloading cycle.

In this regard, it is illustrative to consider how the stresses on the different microstructural elements of the fiber may vary during the unloading step. It may be assumed that the crystalline phase behaves as an elastic material, which implies that the stresses decrease through a moderate reduction in the deformation and, perhaps, through the reorientation of the nanocrystals. Consequently, the viscoelastic behavior is not expected to play a significant role in the mechanical characteristics of this phase. In contrast, the chains that form the hidden length at a given instant will tend to relax in accordance with their elastomeric behaviors. The combination of an elastomeric phase and a viscous (dissipative) medium establishes the conditions for any material to exhibit viscoelasticity [61], and may be identified through the characteristic hysteresis observed in this type of materials when subjected to unloading-reloading cycles [62]. A scheme of the interplay between the different microstructural elements during the loading-unloading cycles is presented in Figure 10.

It must be mentioned, however, that other microstructural features at the nano- or micrometer scales, such as the mesoscale organization of the crystalline and amorphous regions [33,63] or the organization of these regions in nanofibrils [64], might also contribute to this mechanical behavior. It should also be acknowledged that the quantitative effect of viscoelasticity in spider silk may be sensitive to the loading and unloading rates that define the tensile testing procedure. In this work, this variable was not assessed, since all tests were performed at a single loading/unloading rate, so that it remains an open question for future works.

After allowing the fiber to relax at a value of true strain of *ε* = 0, those segments of the chains initially included in the hidden length that do not participate in the changes undergone by the crystalline phase are available for a new loading step. The energy absorbed in this new cycle, however, may be diminished with respect to the previous cycle if irreversible changes were introduced in the crystalline phase in the latter. Although the term ‘irreversible‘ is employed to indicate the transfer of the protein from the hidden length to the crystalline phase, this process is reverted through supercontraction [16,46,65]. It may be hypothesized that the hydrogen network formed between those segments that comprise the amorphous phase plays a critical role in stabilizing the changes in the crystalline phase induced by stretching the material in air. In this regard, it was found that MAS fibers subjected to loading-unloading cycles in water exhibit essentially a full recovery of their tensile properties [66], but the nanocrystals are not affected by immersing the fiber in water [67].

## 5. Conclusions

The analysis of the evolution in the mechanical performance of MAS fibers spun by *A. bruennichi* spiders, after loading and unloading, allows for the obtaining of relevant information on the design of the material and on the deformation micromechanisms involved in their tensile behaviors. The main results obtained from this analysis may be summarized in the four following items:

(1) The true stress-true strain curve beyond a given value of true strain is independent from the previous loading history of the sample.
(2) The elastic modulus measured in the loading steps increases monotonously with increasing values of true strain reached in the cycles.
(3) In contrast, a marginal variation is found in the values of the yield stress measured in the different cycles.
(4) The ability of spider silk to absorb and dissipate energy is increased if the material is tested following a series of loading-unloading cycles in comparison with the values found in a conventional tensile test. Previous studies show, however, that this irreversible effect can be reverted if the fiber is immersed in water and allowed to supercontract.

## Data Availability

Data are available upon request to the corresponding authors.

## Supplementary Materials

The following supporting information can be downloaded at: https://www.mdpi.com/article/10.3390/biomimetics8020164/s1, Figure S1: Examples of SEM micrographs employed to measure the cross sectional area of the fibers; Figure S2: (a) Full set of loading-unloading cycles performed on a single maximum supercontracted MAS fiber. These data were used in the elaboration of this manuscript; (b) Full set of loading-unloading cycles performed on a second single maximum supercontracted MAS fiber; (c) Full set of loading-unloading cycles performed on a third maximum supercontracted MAS fiber; Figure S3: Unloading-reloading curves presented in Figure 4 represented as engineering stress-engineering strain; Figure S4: Determination of the effective *α** parameter of the envelope curve shown in Figure 4.
