# Supplementary material for: Variation in the Elastic Modulus and Increased Energy Dissipation Induced by Cyclic Straining of Argiope bruennichi Major Ampullate Gland Silk
Source: Biomimetics (Basel). 2023 Apr 18;8(2):164. doi: 10.3390/biomimetics8020164 (PMC10123757; doi:10.3390/biomimetics8020164)
Supplement: Supplementary file 1 [file biomimetics-08-00164-s001.zip › biomimetics-2288967-supplementary.pdf]

Article

Variation in the Elastic Modulus and Increased Energy Dissipation Induced by Cyclic Straining of *Argiope bruennichi* Major Ampullate Gland Silk

Ping Jiang [1,*], Li-hua Wu [2], Meng-lei Hu [1], Si-si Tang [2], Zhi-min Qiu [1], Tai-yong Lv [3], Manuel Elices [4], Gustavo V. Guinea [4,5,6,7], and José Pérez-Rigueiro [4,5,6,7,*]

[1] Key Laboratory for Biodiversity Science and Ecological Engineering, Institute of Eco-environment and Resources, College of Life Sciences, Jinggangshan University, Ji'an 343009, China; hml0918@aliyun.com (M.-l.H.); 18870702923@aliyun.com (Z.-m.Q.)
[2] Institute of Qinghai-Tibetan Plateau, Southwest Minzu University, Chengdu 610041, China; 9920090025@jgsu.edu.cn (L.-h.W.); 137336285@aliyun.com (S.-s.T.)
[3] Department of Nuclear Medicine, Affiliated Hospital in Southwest Medical University, Sichuan Key Laboratory of Nuclear Medicine and Molecular Imaging, Luzhou 646000, China; tylv@swmu.edu.cn
[4] Departamento de Ciencia de Materiales, ETSI Caminos, Canales y Puertos, Universidad Politécnica de Madrid, 28040 Madrid, Spain; melices@upm.es (M.E.); gustavovictor.guinea@ctb.upm.es (G.V.G.)
[5] Biomedical Research Networking Center in Bioengineering, Biomaterials and Nanomedicine (CIBER-BBN), 28029 Madrid, Spain
[6] Biomaterials and Regenerative Medicine Group, Instituto de Investigación Sanitaria del Hospital Clínico San Carlos (IdISSC), C/Prof. Martín Lagos s/n 28040 Madrid, Spain
[7] Center for Biomedical Technology (CTB), Universidad Politécnica de Madrid, Pozuelo de Alarcón, Madrid 28223, Spain
* Correspondence: jiangping@jgsu.edu.cn (P.J.); jose.perez@ctb.upm.es (J.P.-R.)

Supplementary

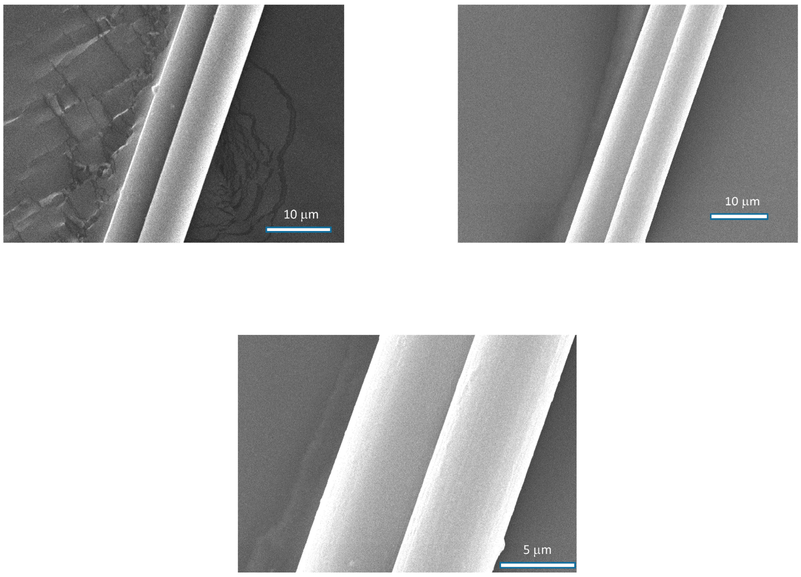

Figure S1. Examples of SEM micrographs employed to measure the cross sectional area of the fibers.

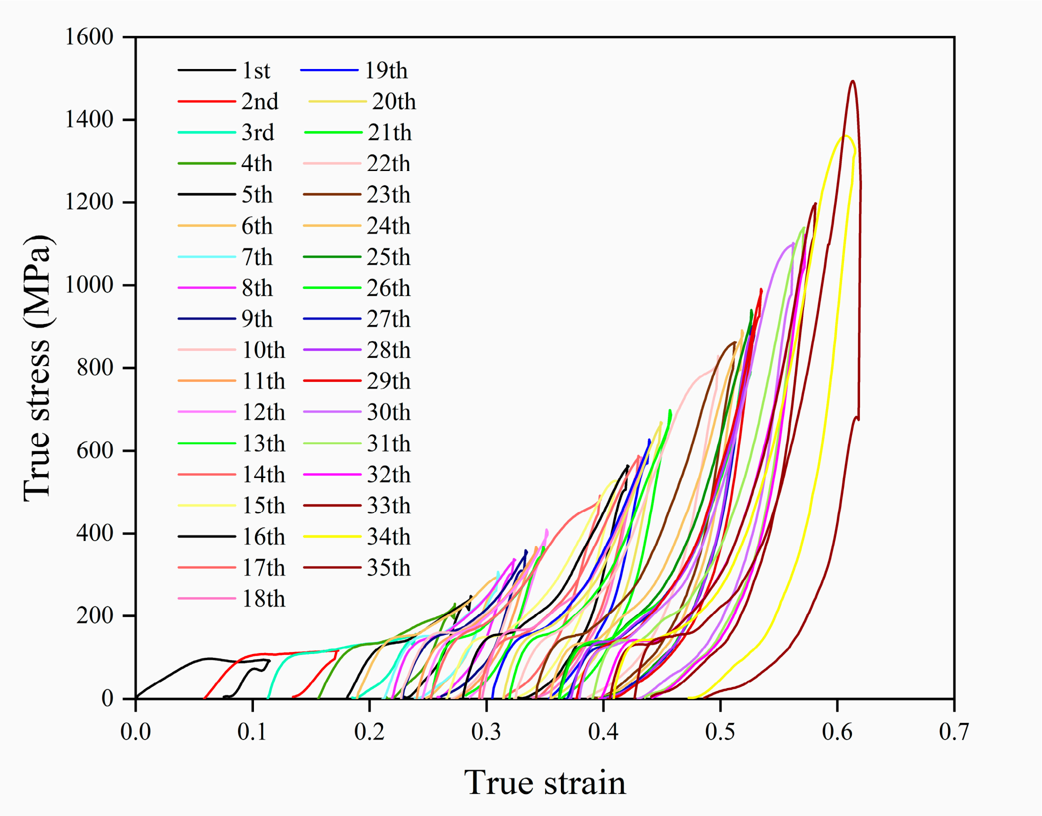

Figure S2. a: Full set of loading-unloading cycles performed on a single maximum su-percontracted MAS fiber. These data were used in the elaboration of this manuscript.

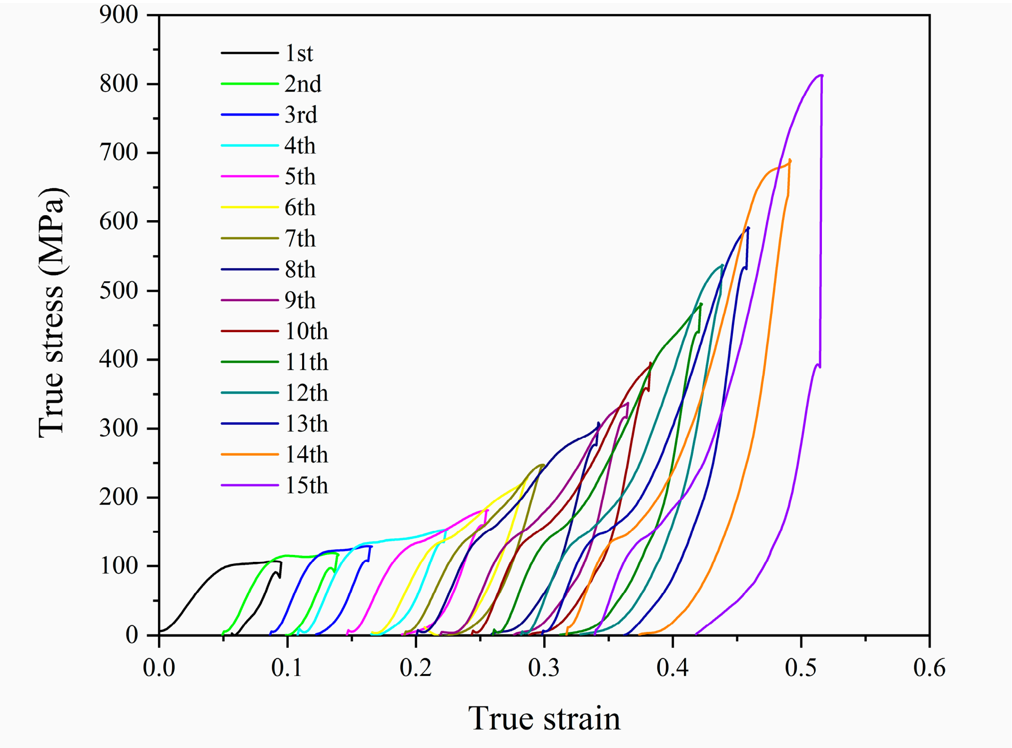

Figure S2. b: Full set of loading-unloading cycles performed on a second single maximum supercontracted MAS fiber.

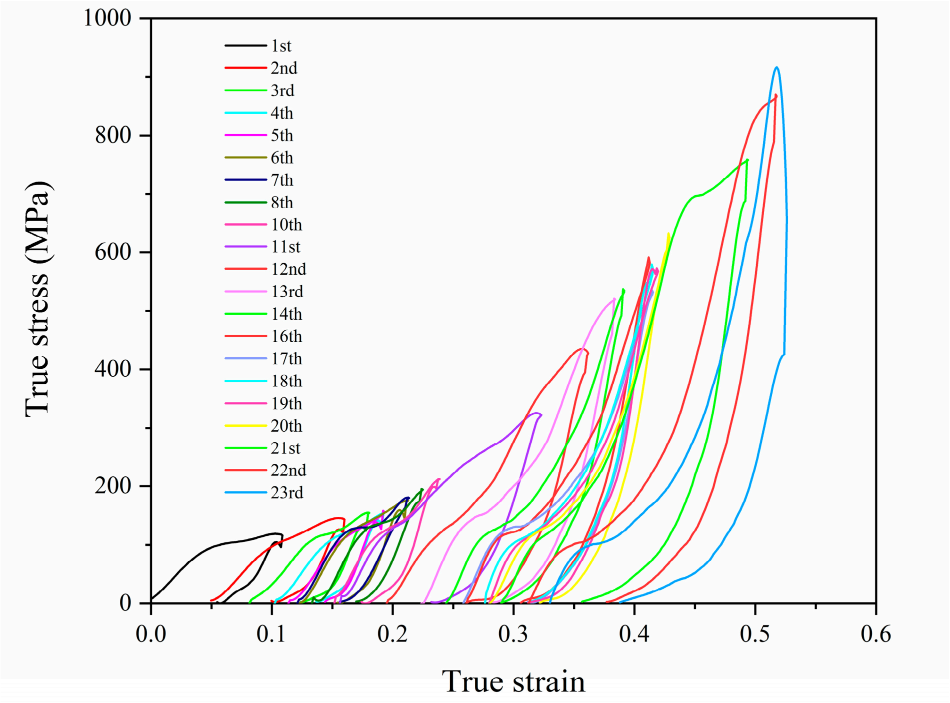

Figure S2. c: Full set of loading-unloading cycles performed on a third maximum su-percontracted MAS fiber.

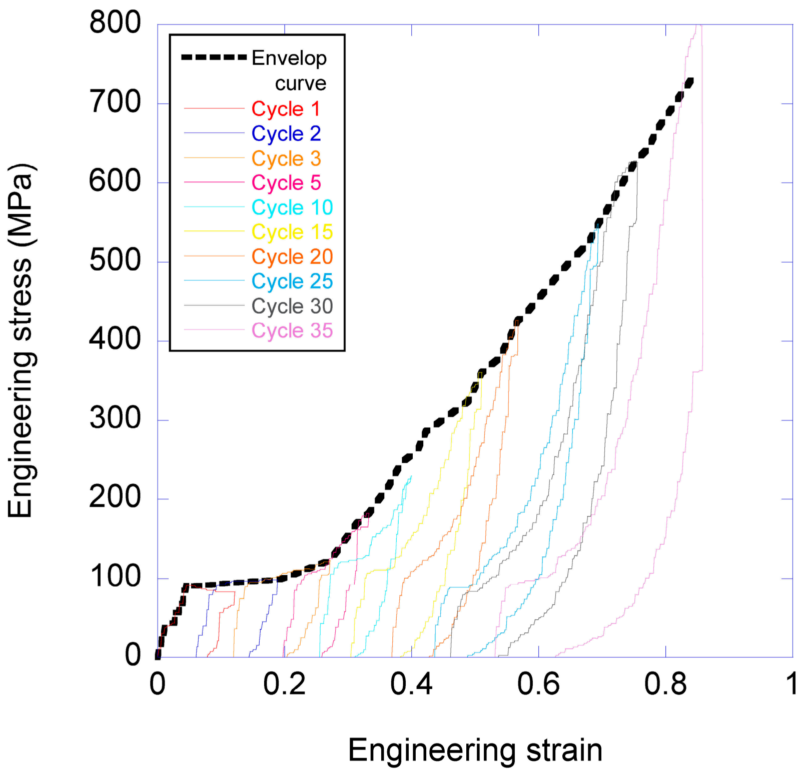

Figure S3. Unloading-reloading curves presented in Figure 4 repre-sented as engineering stress-engineering strain;.

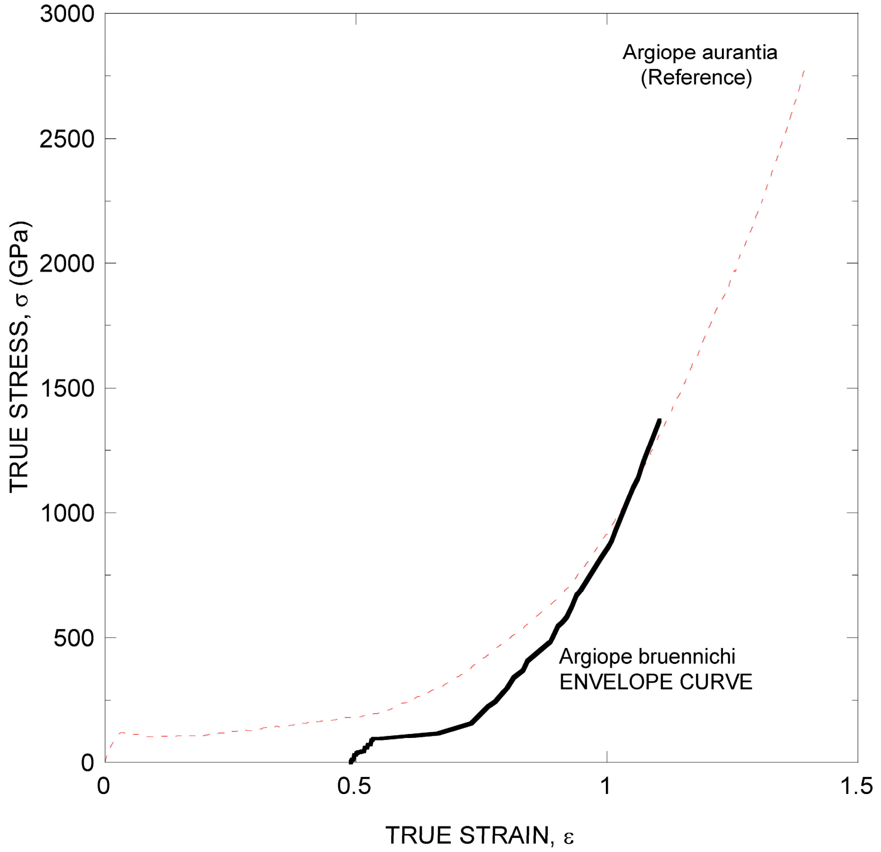

Figure S4. Determination of the effective * pa-rameter of the envelope curve shown in Figure 4.
