# Supplementary figures and images for: Variation in the Elastic Modulus and Increased Energy Dissipation Induced by Cyclic Straining of Argiope bruennichi Major Ampullate Gland Silk
Source: Biomimetics (Basel). 2023 Apr 18;8(2):164. doi: 10.3390/biomimetics8020164 (PMC10123757; doi:10.3390/biomimetics8020164)

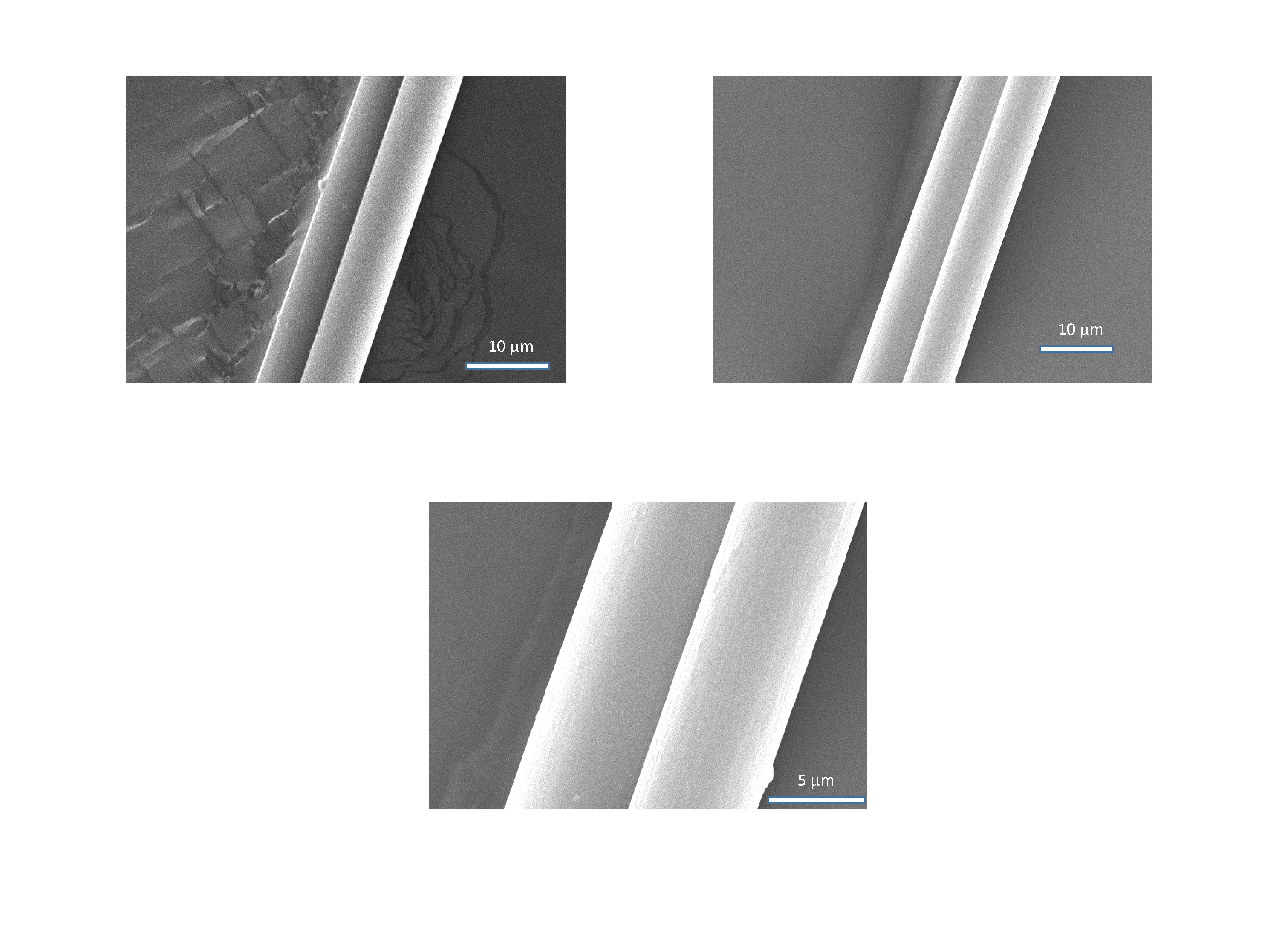

Supplement: Supplementary file 1 [file biomimetics-08-00164-s001.zip › Supplementary Figure 1_SEM micrographs.jpg]

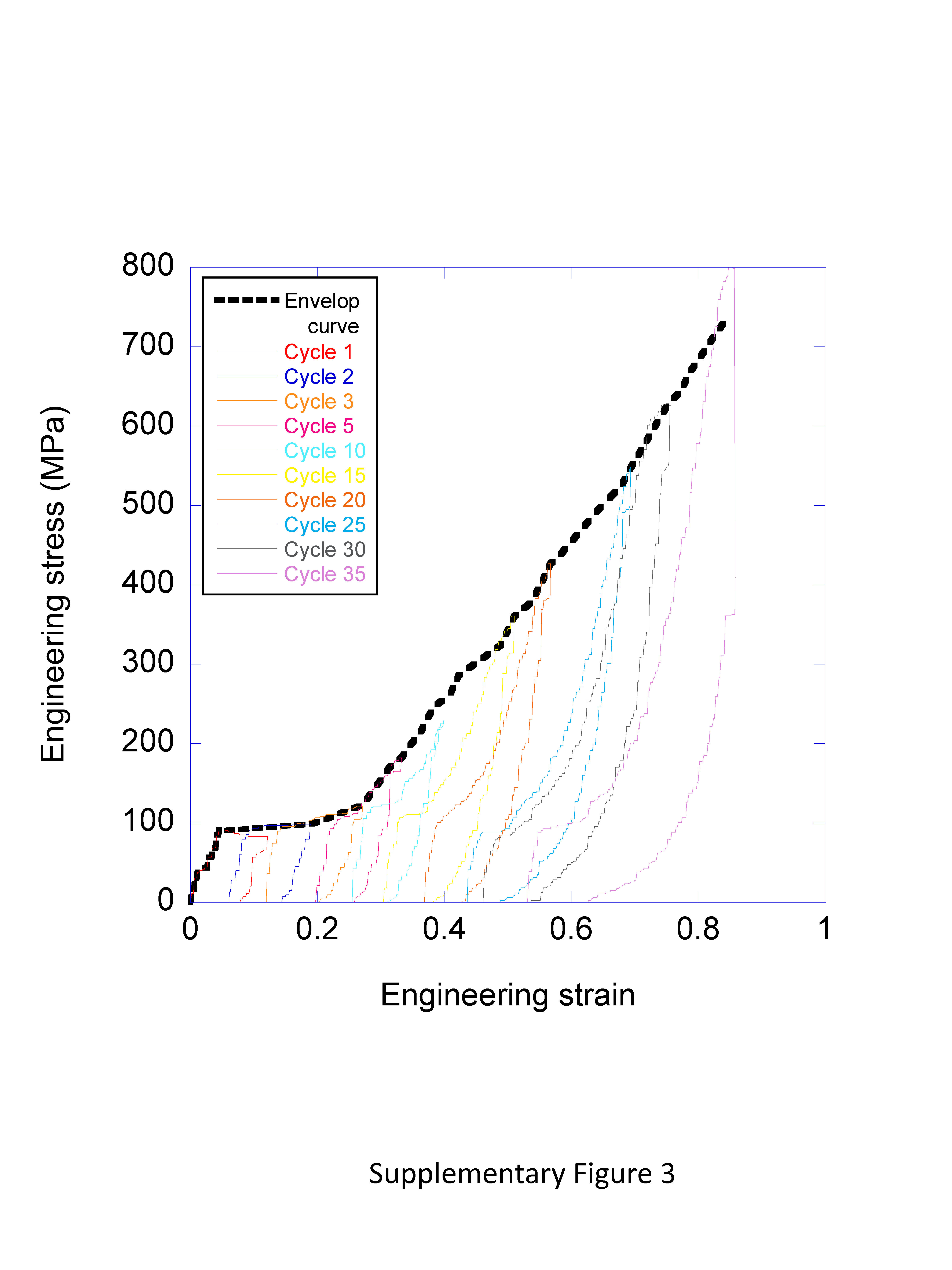

Supplement: Supplementary file 1 [file biomimetics-08-00164-s001.zip › Supplementary Figure 3.jpg]

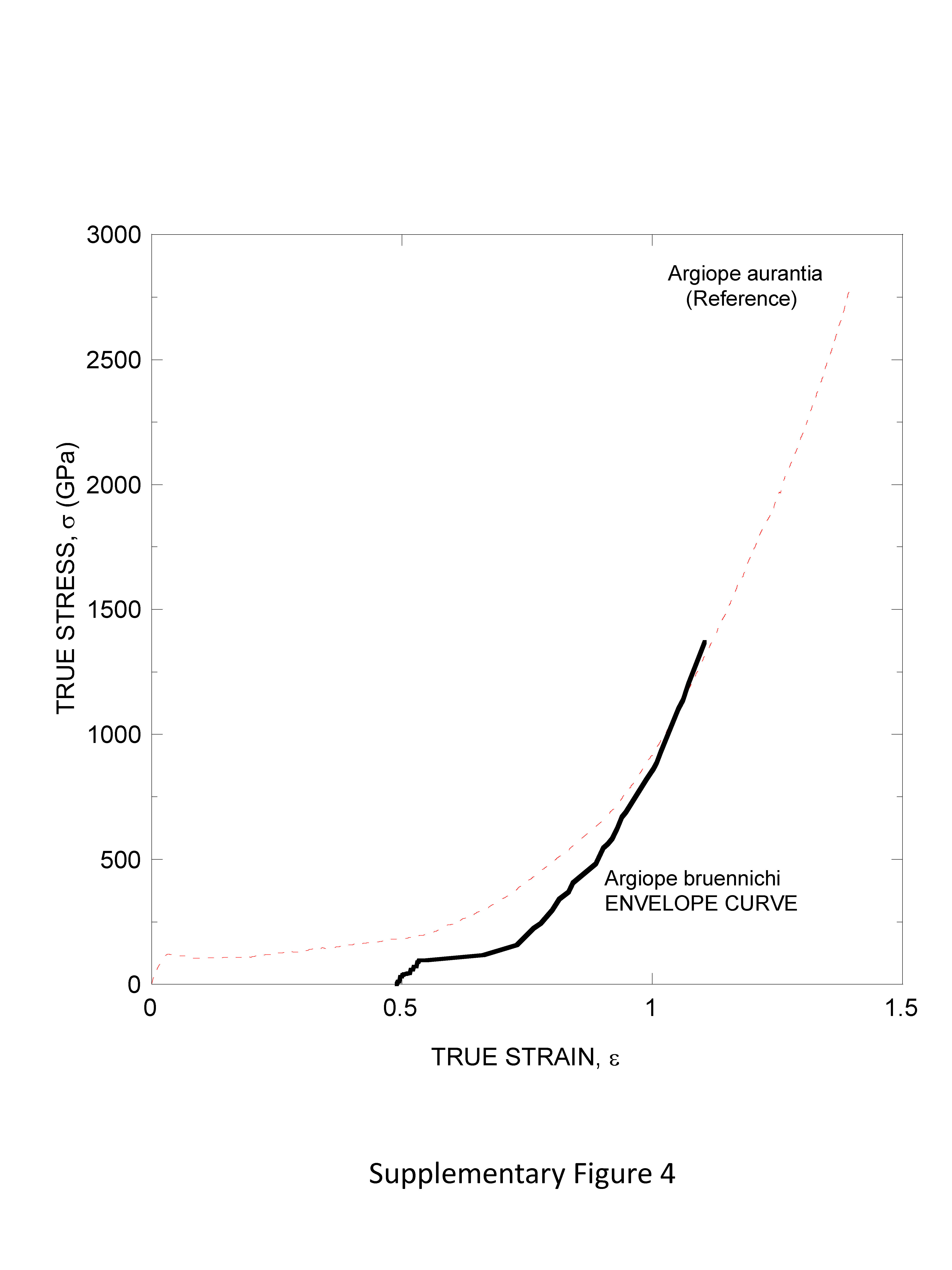

Supplement: Supplementary file 1 [file biomimetics-08-00164-s001.zip › Supplementary Figure 4.jpg]
